# The *Arabidopsis *translocator protein (*At*TSPO) is regulated at multiple levels in response to salt stress and perturbations in tetrapyrrole metabolism

**DOI:** 10.1186/1471-2229-11-108

**Published:** 2011-06-20

**Authors:** Emilia Balsemão-Pires, Yvon Jaillais, Bradley JSC Olson, Leonardo R Andrade, James G Umen, Joanne Chory, Gilberto Sachetto-Martins

**Affiliations:** 1 Laboratório de Genômica Funcional e Transdução de Sinal, Departamento de Genética, Universidade Federal do Rio de Janeiro, Rio de Janeiro, Brazil; 2 Plant Biology Laboratory, The Salk Institute, 10010 North Torrey Pines Road, La Jolla, CA 92037, USA; 3 Howard Hughes Medical Institute 4000 Jones Bridge RoadChevy Chase, MD 20815-6789, USA; 4 Laboratório de Biomineralização, Instituto de Ciências Biomédicas, Universidade Federal do Rio de Janeiro, Brasil

**Keywords:** plant TSPO, subcellular localization, abiotic stress, regulation, chloroplast

## Abstract

**Background:**

The translocator protein 18 kDa (TSPO), previously known as the peripheral-type benzodiazepine receptor (PBR), is important for many cellular functions in mammals and bacteria, such as steroid biosynthesis, cellular respiration, cell proliferation, apoptosis, immunomodulation, transport of porphyrins and anions. *Arabidopsis thaliana *contains a single *TSPO/PBR*-related gene with a 40 amino acid N-terminal extension compared to its homologs in bacteria or mammals suggesting it might be chloroplast or mitochondrial localized.

**Results:**

To test if the TSPO N-terminal extension targets it to organelles, we fused three potential translational start sites in the *TSPO *cDNA to the N-terminus of GFP (*At*TSPO:eGFP). The location of the *At*TSPO:eGFP fusion protein was found to depend on the translational start position and the conditions under which plants were grown. Full-length *At*TSPO:eGFP fusion protein was found in the endoplasmic reticulum and in vesicles of unknown identity when plants were grown in standard conditions. However, full length *At*TSPO:eGFP localized to chloroplasts when grown in the presence of 150 mM NaCl, conditions of salt stress. In contrast, when *At*TSPO:eGFP was truncated to the second or third start codon at amino acid position 21 or 42, the fusion protein co-localized with a mitochondrial marker in standard conditions. Using promoter *GUS *fusions, qRT-PCR, fluorescent protein tagging, and chloroplast fractionation approaches, we demonstrate that *At*TSPO levels are regulated at the transcriptional, post-transcriptional and post-translational levels in response to abiotic stress conditions. Salt-responsive genes are increased in a *tspo-1 knock-down *mutant compared to wild type under conditions of salt stress, while they are decreased when *At*TSPO is overexpressed. Mutations in tetrapyrrole biosynthesis genes and the application of chlorophyll or carotenoid biosynthesis inhibitors also affect *AtTSPO *expression.

**Conclusion:**

Our data suggest that AtTSPO plays a role in the response of *Arabidopsis *to high salt stress. Salt stress leads to re-localization of the AtTSPO from the ER to chloroplasts through its N-terminal extension. In addition, our results show that *AtTSPO *is regulated at the transcriptional level in tetrapyrrole biosynthetic mutants. Thus, we propose that *At*TSPO may play a role in transporting tetrapyrrole intermediates during salt stress and other conditions in which tetrapyrrole metabolism is compromised.

## Background

Higher plants synthesize four major tetrapyrroles (chlorophyll, haem, sirohaem and phytochromobilin) via a common branched pathway [1-3] (Additional file 1). In metazoans, heme and siroheme are synthesized in mitochondria, but in plants tetrapyrrole biosynthesis is plastid-localized, suggesting that tetrapyrroles are transported from the chloroplast to the mitochondria. This suggests that late stages of the heme biosynthetic pathway are present in both chloroplasts and mitochondria (Additional file 1). The concentration of tetrapyrrole intermediates is tightly controlled because these compounds are photoreactive and can generate reactive oxygen species (ROS). Additionally, many of the enzymes in this pathway are regulated by environmental stimuli and development signals [4,5].

In mammals, an 18-kDa peripheral-type benzodiazepine receptor (TSPO/PBR) is localized in the outer mitochondrial membrane [6] where it binds other proteins, such as the 34-kDa voltage-dependent anion channel and the inner membrane adenine nucleotide carrier [7]. TSPO was originally named the "peripheral benzodiazepine receptor" (PBR), however, it has more recently been renamed "TSPO" reflecting its structural and functional similarity to the bacterial tryptophan-rich sensory protein [8].

TSPO primarily functions to transport heme, porphyrins, steroids and anions [8-11]. However, TSPO proteins are also important for cellular respiration [12], cell proliferation [13] and apoptosis [14]. For example, in erythroids, in response to stress, TSPO is important for transporting porphyrins, which induce the expression of heme biosynthesis genes. Likewise, in mouse erythroleukemia cells TSPO has been shown to transport protoporphyrin IX playing a key role in tetrapyrrole and heme biosynthesis [15].

In the α-proteobacterium *Rhodobacter sphaeroides *TSPO is localized in the outer membrane and its expression is induced by oxygen [16]. Under conditions of high oxygen, TSPO negatively regulates the expression of photosynthetic genes by exporting excess intermediates of the tetrapyrrole pathway, such as Mg-Protoporphyrin IX (Mg-ProtoIX) and MgProtoIX Monomethyl ester [17]. The rat *TSPO *homologue complements the *Rhodobacter tspo *mutant, suggesting that the function of TSPO is conserved in *R. sphaeroides *and metazoans [18].

Evidence for a functional TSPO protein in *Arabidopsis thaliana *and other plants has been previously reported [19]. Transport studies with the recombinant *Arabidopsis *TSPO in *Escherichia coli *revealed a benzodiazepine-stimulated high-affinity uptake of protoporphyrin and cholesterol, leading to the hypothesis that the *Arabidopsis *homologue functions in the transport of protoporphyrinogen IX to the mitochondria where heme can be synthesized. However, the role of *At*TSPO in plant metabolism is still unknown.

In animals and yeast, TSPO is found in the outer membrane of the mitochondria [6,20]. However the localization of TSPO in plants remains controversial. Lindenman *et al*. [19] used immunogold staining to show that TSPO is localized in the outer membrane of plastids and mitochondria in *Digitalis lanata *leaves. However, follow up Western blot experiments could only detect TSPO in mitochondrial fractions. In a separate study, TSPO was found in nuclear fractions prepared from *Solanum tuberosum *meristematic tissues, while low levels were detected in chloroplast fractions [21]. In *Physcomitrella patens*, transient expression of TSPO fused to the N-terminus of GFP, PpTSPO:GFP, localized to the mitochondria [22]. In *Arabidopsis*, fusion of TSPO to the C-terminus of YFP resulted in YFP:TSPO being found in the endoplasmic reticulum and the Golgi stacks [23].

The *Arabidopsis *genome contains a single *TSPO*-related gene (*AtTSPO*). The predicted protein shares a high degree of similarity to the central domain of its bacterial and mammalian homologs. However, *At*TSPO has a 40 amino acid N-terminal extension that is not present in either bacteria or mammals. Moreover, within these 40 amino acids are three in-frame ATG-codons that could code for the first methionine (at positions M1, M21 and M42) (Additional file 2) [19]. To determine whether this region contains organellar targeting information, we developed a series of fusion proteins using the 3 different start sites. Our results demonstrate that *At*TSPO was found in different organellar compartments depending on environmental stress. These results, along with analysis of an insertional mutation and expression studies, show that *At*TSPO plays an important role in allowing *Arabidopsis *to cope with high salt stress.

## Results

### Induction *AtTSPO *gene expression by abiotic stress

In *Physcomitrella patens*, the expression of *PpTSPO-1 *is induced by salt stress and abscisic acid (ABA) [22]. *AtTSPO *is also induced by salt stress in *Arabidopsis *[24], as well as in *Arabidopsis *cell cultures [23]. We further defined the transcript abundance of *AtTSPO *in 5-day old seedlings treated with NaCl, mannitol, ABA and methyl viologen (MV), by extracting total RNA from these plants and performing quantitative real-time PCR (qRT-PCR).

**Figure 1:**
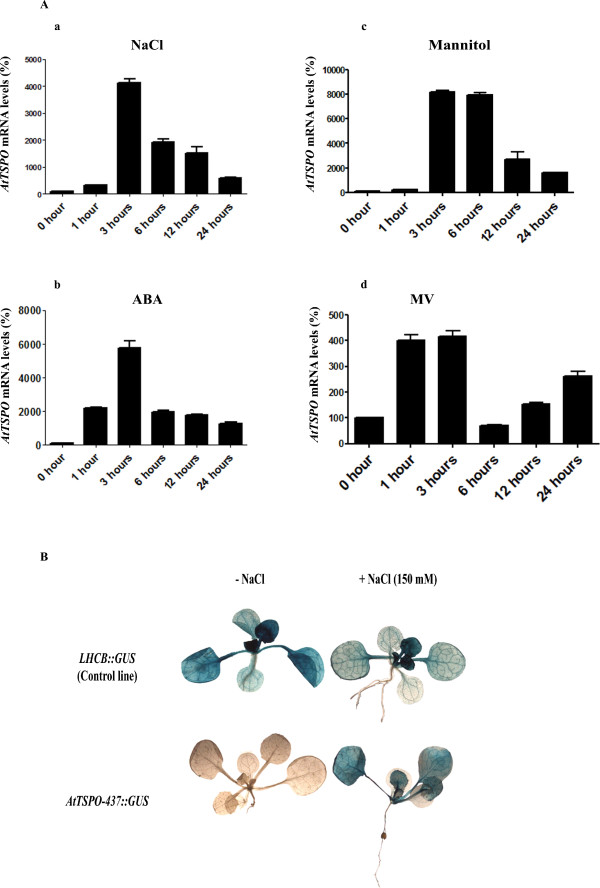
**Induction of *AtTSPO *mRNA by abiotic stresses**. (**A**) Quantitative real-time PCR analyses of *AtTspO *transcripts upon treatment of different stresses, (a) 150 mM NaCl, (b) 1 μM ABA, (c) 250 mM mannitol and (d) 0.2 μM methyl viologen. Relative expression levels were calculated and *ACTIN *(*At*3g18780) and *18S *rRNA (*At*3g41768) here used as reference genes. (**B**) GUS expression in *AtTSPO*-437::*GUS *and *LHCB::GUS *lines in 15-day-old transgenic *Arabidopsis *plants either untreated or treated with 150 mM NaCl.

Compared to untreated plants, 150 mM NaCl, 250 mM mannitol, 1 μM ABA and 0.2 μM methyl viologen (MV) resulted in increased *AtTSPO *expression (Figure 1A). The kinetics of *AtTSPO *induction by NaCl and ABA stress were similar, peaking 3 hours after treatment and slowly decreasing between 6-25 h (Figure 1A-a and 1A-b respectively). Addition of mannitol resulted in peak *AtTSPO *expression between 3-6 h and then slowly decreased in abundance (Figure 1A-a, 1A-b and 1A-c). However mannitol treatment showed a two-fold induction compared to treatment with NaCl for 3 h (Figure 1A-a and 1A-c), which suggests that *AtTSPO *is induced by osmotic stress rather than salt stress. *At*TSPO is rapidly induced by MV treatment, showing induction at 1 h, peaking by 3 h and then falling to basal levels within before increasing between 12-24 h (Figure 1A-d).

To determine if *AtTSPO *accumulation was a transcriptional response to NaCl stress, a construct, containing 437 bp upstream the putative translational start site of the *AtTSPO *gene was fused to the *uidA *reporter gene (*AtTSPO*-437::*GUS*), and transformed into plants, allowing *in vivo *analysis of *AtTSPO *transcriptional response to stress conditions. *AtTSPO*-437::*GUS *was found to be induced by 150 mM NaCl within 3 h of treatment, which is similar to qRT-PCR results of the endogenous gene (Figure 1B). In control experiments, 150 mM NaCl resulted in a small decrease of expression of *LHCB::GUS *(Figure 1B). Together these results suggest that the 437 bp region of *AtTSPO *promoter is sufficient for transcriptional regulation of TSPO.

### Identification and characterization of *AtTSPO *mutants

**Figure 2:**
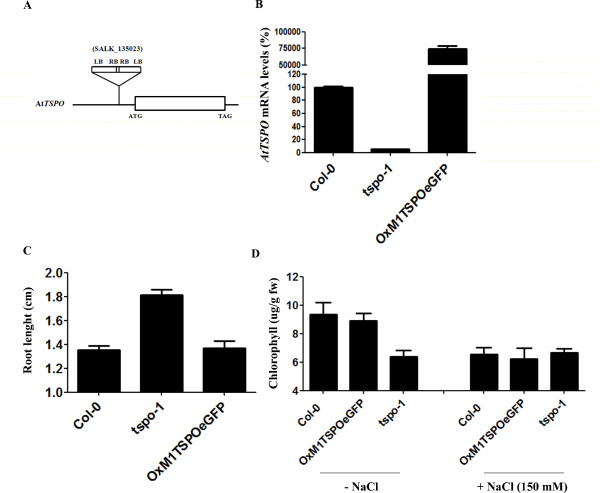
**Phenotype of mutants with different levels of *AtTSPO *expression**. (**A**) Schematic representation of isolated insertional mutant of *AtTSPO *in *Arabidopsis*. Two copies of the T-DNA were inserted in tandem 123 bp upstream from the translational initiation codon of *AtTSPO*. (**B**) Total RNA was isolated from 5 day-old seedlings, reverse-transcribed and subjected to qRT-PCR. Data shown represent mean values obtained from independent amplification reactions (n = 3) and biological replicates (n = 2). Bars represent the standard error of biological replicates. (**C**) Root lengths of at least 100 individual 7-day-old seedlings grown in 16 h photoperiods. (**D**) Chlorophyll concentrations in 14-day-old, in vitro-grown plants of the indicated genotypes were determined spectrophotometrically. Values shown are means derived from three independent samples, each sample containing 100 mg of fresh weight. Units are μg of chlorophyll a + b per g of fresh weight (fw).

To determine the function of *AtTSPO in vivo*, we obtained a T-DNA insertional mutant (SALK_135023) [25] in *AtTSPO*. This line (*tspo-1*) was found to have two tandem T-DNA insertions, 123 bp upstream from the translational initiation codon of the *AtTSPO *gene (Figure 2A). Homozygous lines were then confirmed to be knock-down mutants by quantitative real time PCR (qRT-PCR) analysis. In this mutant, *TSPO *mRNA levels are about 20% of wild type (Figure 2B).

*AtTSPO *fused or not to the N-terminus of GFP was constitutively overexpressed from the CaMV 35S promoter in transgenic *Arabidopsis *lines (OxM1TSPO and OxM1TSPO:eGFP). We obtained 10 over-expression lines, but focused on the two homozygous lines that exhibited ~500 fold over-expression of *AtTSPO *(Figure 2B). The *tspo-1*, OxM1TSPO:eGFP and wild-type lines were grown side-by-side on either Murashige & Skoog (MS) agar medium or soil, and were monitored for possible abnormal phenotypes. The knock-down plants had longer roots compared to the wild type and the over-expression lines (Figure 2C). Moreover, *tspo-1 *accumulated ~30% less chlorophyll than either the wild type or the overexpression lines in the presence of 150 mM NaCl (Figure 2D).

### The expression of stress-response genes is enhanced in *tspo-1*

**Figure 3:**
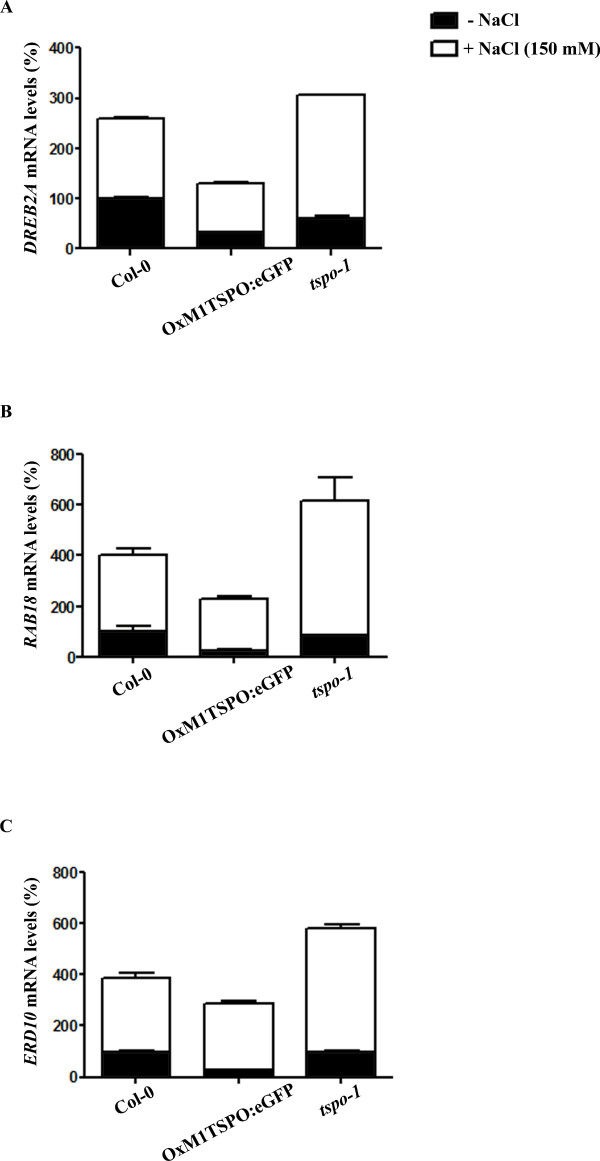
**Stress-response genes are up-regulated in *tspo-1 *during salt stress**. (**A**)-(**C**) Stress-induced gene expression in OxM1TSPO:eGFP and *tspo-1 *lines compared to wild type plants, by qPCR. 5-day-old seedlings grown under standard conditions and transferred for 3 hours to plates containing 150 mM NaCl. (**A**) *DREB2A*, (**B**) *RAB18 *and (**C**) *ERD10 *mRNA levels were determined by quantitative qRT-PCR. Relative amounts were calculated and normalized relative to Col-0 non-treated (100%). The *ACTIN *and *18S *rRNA were used as reference genes. *ACTIN, At*3g18780; *18S *RNA, *At*3g41768; *RAB18*, *At*5g66400; *ERD10*, *At*1g20450; *DREB2A*, *At*5g05410. Data shown represent mean values obtained from independent amplification reactions (n = 3) and biological replicates (n = 2). Relative expression levels were calculated. Bars represent the standard error of biological replicates.

*AtTSPO *expression was previously shown to be regulated by osmotic stress in germination and seedling growth assays [23]. Because TSPO regulates the expression of photosynthetic genes in *R. sphaeroides *[16], we hypothesized that *tspo-1 *or OxM1TSPO:eGFP mutants might have an impaired salt stress response. We examined the expression of some well-known salt stress-regulated genes (RAB18, ERD10 and DREB2A) [26]. As expected, stress marker genes were induced by 150 mM NaCl in wild-type plants (Figure 3A, B and 3C). In *At*TSPO over-expression lines, the levels of DREB2A and RAB18 were lower but no significant change ERD10 expression was observed (Figure 3A, B and 3C).

In *tspo-1 *mutants, 3 h of 150 mM NaCl treatment resulted in the increased expression of all three stress marker genes (Figure 3A, B and 3C). Taken together these results show that *AtTSPO *plays an important role in regulating the expression of stress response genes.

### Expression of light-regulated tetrapyrrole genes are repressed in the *tspo-1 *knock-down mutant

Consistent with TSPO transporting tetrapyrroles [17,19,27], *tspo-1 *plants accumulated less chlorophyll than wild-type plants (Figure 2D). Because we found that TSPO is involved in the salt stress response and because TSPO negatively regulates photosynthetic genes in *R. sphaeroides *[17]. We next analyzed the expression of a few key chlorophyll biosynthesis genes in *tspo-1 *plants.

**Figure 4:**
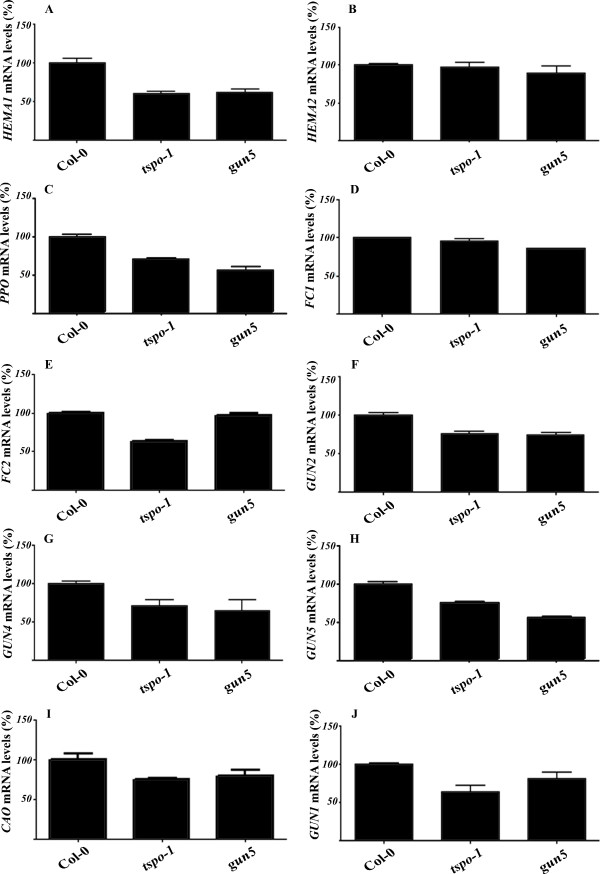
**Expression of tetrapyrrole biosynthesis genes in *tspo-1 *mutant**. qRT-PCR analyses of tetrapyrrole biosynthesis genes in *Col-0*, *tspo-1 *and *gun5 *5-days-old seedlings grown in constant light. Relative amounts were calculated and normalized relative to *Col-0 *non-treated (100%). With the exception of *HEMA2 *and *FC1*, all the genes have been show to be regulated by light. The data are presented following the enzymes order in the tetrapyrrole biosynthesis. The *ACTIN *and *18S *rRNA genes were used as control. *ACTIN*, *At*3g18780; *18S *rRNA, *At*3g41768; (**A**) *HEMA1 *(Glutamyl-tRNA reductase 1 - *At*1g58290) (**B**) *HEMA2 *(Glutamyl-tRNA reductase 2 - *At*1g04490); (**C**) *PPO *(Protoporphyrinogen oxidase *- At*4g01690); (**D**) *FC1 *(Ferrochelatase 1 - *At*5g26030); (**E**) *FC2 *(Ferrochelatase 2 - *At*2g30390); (**F**) *GUN2 *(Heme oxygenase 1 - *At*2g26670); (**G**) *GUN4 *(Regulator of Mg-porphyrin synthesis - *At*3g59400); (**H**) *GUN5 *(Mg-chelatase subunit H - *At*5g13630); (**I**) *CAO *(Chlorophyllide A oxygenase - *At*1g44446); and (**J**) *GUN1 *(Pentatricopeptide repeat (PPR) protein - *At*2g31400). Data shown represent mean values obtained from independent amplification reactions (n = 3) and biological replicates (n = 2). Relative expression levels were calculated. Bars represent the standard error of biological replicates.

Initially, we determined the mRNA levels of most of the key genes in the tetrapyrrole pathway (Additional file 1) in *tspo-1 *and *gun5 *mutants. *GUN5 *encodes the H subunit of chloroplastic Mg-chelatase, which is involved in the perception of altered levels of tetrapyrrolic intermediates [28]. All tetrapyrrole biosynthetic genes known to be light-dependent [29] were found to be down-regulated in *tspo-1*, as well as in *gun5 *mutants [28] (Figure 4A, C, E, F, G and 4H), whereas the expression of the two light-independent genes were unaffected in wild-type and *tspo-1 *mutant (Figure 4B and 4D).

### Correlation of tetrapyrrole pathway flux and *AtTSPO *mRNA levels

**Figure 5:**
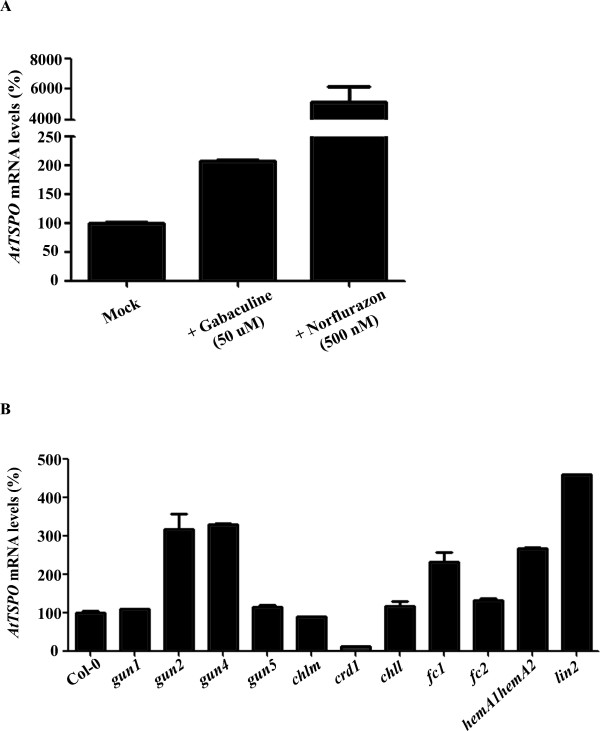
**Relationship between tetrapyrrole flux and *AtTSPO *expression**. (**A**) *AtTSPO *expression in wild-type plants germinated in 50 μM of gabaculine or 500 nM of norflurazon compared to untreated plants. (**B**) *AtTSPO *mRNA levels in different mutants of the tetrapyrrole pathway. Relative amounts were calculated and normalized relative to Col-0 non-treated (100%). The *ACTIN *and *18S *rRNA genes were used as control. *ACTIN*, *At*3g18780; *18S *RNA, *At*3g41768. Data shown represent mean values obtained from independent amplification reactions (n = 3) and biological replicates (n = 2). Relative expression levels were calculated. Bars represent the standard error of biological replicates.

*tspo-1 *mutants present reduced levels of light-regulated tetrapyrrole metabolism genes (Figure 4A, C, E, F, G, and 4H) and also have low chlorophyll content (Figure 2D). In order to investigate if decreasing flux of tetrapyrrole intermediates would affect *AtTSPO *expression in wild-type plants, we used two different drugs that interfere with tetrapyrrole biosynthesis, Gabaculine and Norflurazon. Gabaculine acts as a tetrapyrrole biosynthesis inhibitor by blocking the glutamate-1-semi aldehyde aminotransferase activity [30,31]. The herbicide Norflurazon inhibits carotenoid biosynthesis and indirectly affects enzymes in tetrapyrrole biosynthesis [32-34]. *AtTSPO *mRNA levels increased 2-fold in plants treated with 50 μM of gabaculine and up to 500-fold after 500 nM norflurazon treatment (Figure 5A).

To explore if *AtTSPO *expression is affected by genetic alterations of the tetrapyrrole biosynthesis pathway, we analyzed the expression of *AtTSPO *in different mutant backgrounds (Additional file 1). We found that *AtTSPO *levels are differently altered in various tetrapyrrole pathway mutants. *AtTSPO *steady-state levels were increased in *gun2 *(allele of *hy1 *- required for phytochromobilin synthesis from heme) [35], *gun4 *(mutant in the Protoporphyrin IX- and Mg-Protoporphyrin IX-binding protein) [36], *fc1 *(mutant in the ferrochelatase) [37], *hemA1hemA2 *double mutant (mutant in both glutamyl-tRNA reductases genes) [38] and *lin-2 *(mutant in the coproporphyrinogen III oxidase) [39] (Figure 5B). The increased expression of *AtTSPO *in these mutants with reduced tetrapyrrole levels is consistent with *At*TSPO transporting tetrapyrroles for roles in other compartments. The only biosynthetic mutant that resulted in reduced *AtTSPO *levels was *crd1 *(mutant in the Mg-protoporphyrin IX monomethyl ester cyclase) [40] (Figure 5B). All these mutations, in exception of *crd1 *[41], inhibit somehow ALA synthesis, suggesting that disturbances in tetrapyrrole biosynthesis or accumulation affect *AtTSPO *mRNA expression.

### *At*TSPO localization depends on the translational start site used

*AtTSPO *(*At*2g47770) encodes a protein with a predicted molecular weight of 18 kDa. This protein has three possible in-frame ATG-start codons (M1, M21 and M42) in its N-terminal extension region (Additional file 2) [19].

Since reports of plant TSPO localization have resulted in different findings subcellular localization of plant TSPO [19,21,23] we re-examined the subcellular location of *At*TSPO and evaluated the roles of the N-terminal extension in targeting *AtTSPO *within the cell. Past studies [20,23] have utilized N-terminal GFP fusions that might block potential organellar targeting of *At*TSPO, particularly mitochondrial or plastid localization. To allow proper targeting of *At*TSPO fusions to GFP, *AtTSPO *was placed on the N-terminus of GFP. Three constructs were made, representing each of the potential start codons M1 (OxM1TSPO:eGFP), M21 (OxM21TSPO:eGFP) and M42 (OxM42TSPO:eGFP) and expressed from the CaMV 35S promoter in *Arabidopsis*.

**Figure 6:**
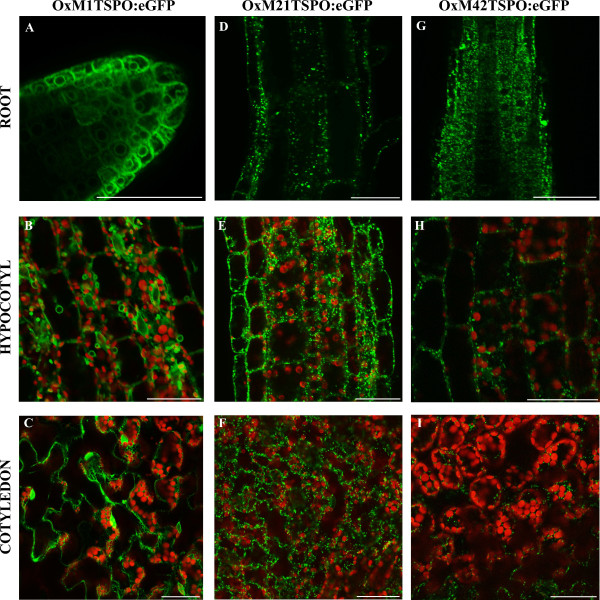
***At*TSPO has different sub-cellular location depending on the translational start site used**. Confocal images of OxM1TSPO:eGFP (**A-C**), OxM21TSPO:eGFP (**D-F**) and OxM42TSPO:eGFP (**G-I**) localization. OxM1TSPO:eGFP localizes in the ER and vesicles of unknown function in the root (**A**), hypocotyl (**B**) and cotyledon (**C**). OxM21TSPO:eGFP localizes in the mitochondria of root (**D**), hypocotyl (**E**) and cotyledon (**F**). OxM42TSPO:eGFP show mitochondria localization in root (**G**), hypocotyls (**H**) and cotyledons (**I**). GFP fluorescence is represented by green and chlorophyll auto fluorescence in red. The samples were incubated with Mitotracker to identify mitochondria (see Additional file 3). Homozygous transgenic plants harboring 35S-TSPO:eGFP in wild-type background were used for the analysis. Scale bars = 50 μm.

*At*TSPO:eGFP subcellular localization was observed in root, hypocotyls and cotyledons of these lines by confocal microscopy. Full-length *At*TSPO:eGFP (OxM1TSPO:eGFP) was found in the endoplasmic reticulum (ER) of the root tip (Figure 6A) and cotyledons (Figure 6C) in five day-old seedlings. However, in the hypocotyls of these plants, the fusion protein was found in the ER and in vesicles of unknown identity (Figure 6B). When M21 (OxM21TSPO:eGFP) or M42 (OxM42TSPO:eGFP) were used, the fusion proteins always co-localized with mitotracker, indicating a mitochondrial localization (Figure 6D, E, F, G, H and 6I) (Additional file 3). These results corroborate the previous observations of mitochondrial localization of TSPO in *D. Lanata *leaves by immunogold staining and in *Arabidopsis *by western blot experiments [19], as well as the endoplasmic reticulum located protein [23], indicating that the alternative use of three initiation codons could be important for *At*TSPO localization and its post-translational control.

### OxM1TSPOeGFP becomes associated with plastids following high salt stress

**Figure 7:**
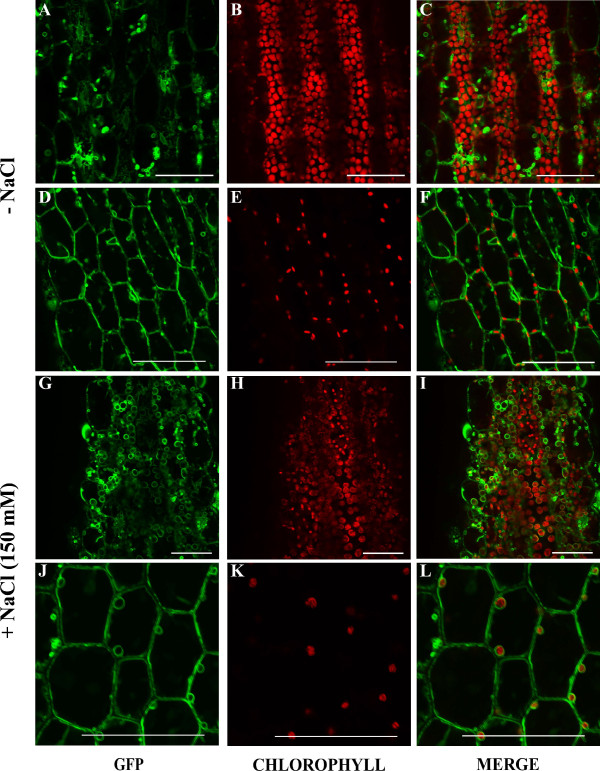
**OxM1TSPOeGFP localizes in chloroplasts upon salt stress**. (**A-F**) Confocal analyses show OxM1TSPO:eGFP localization in the ER and vesicles of unknown function in hypocotyls of 5-day-old seedlings grown in the standard conditions. (**G-L**) Confocal analyses show OxM1TSPO:eGFP chloroplast localization in hypocotyls of 5-day-old seedlings grown in the presence of 150 mM NaCl. GFP fluorescence channel is represented in green and chlorophyll auto fluorescence channel is represented in red. Homozygous transgenic plants harboring 35S-TSPO:eGFP in wild-type background were used for the analysis. Scale bars = 50 μm.

Having established a key role for *At*TSPO in response to abiotic stress, we next examined the localization of *At*TSPO:eGFP fusion proteins in plants subjected to various stress conditions. 5 day-old seedlings were treated with 250 mM mannitol, 1 μM ABA, 0.2 μM MV and 150 mM NaCl. After 18 hours of treatment, OxM1TSPO:eGFP became localized to the plastid (Figure 7G, H, I, J, K and 7L), while neither OxM21TSPO:eGFP nor OxM42TSPO:eGFP had altered localization even with 5 day extended NaCl treatment (data not shown). *At*TSPO:GFP localization did not change when plants were treated with mannitol, ABA or MV (data not shown).

**Figure 8:**
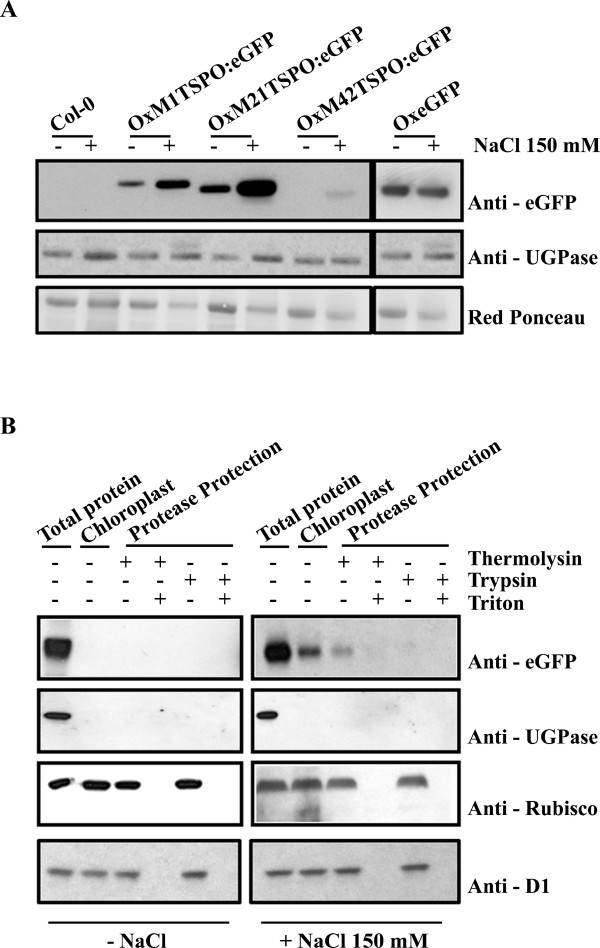
***At*TSPO accumulation and chloroplast localization upon salt stress**. (**A**) Immunoblot analysis of Ox*At*TSPO:eGFP (OxM1TSPO:eGFP, OxM21TSPO:eGFP and OxM42TSPO:eGFP) fusion proteins detected in plants with an antibody to GFP. Plants were untreated, or treated with 150 mM NaCl. As a control wild-type plants and plants over-expressing GFP (OxeGFP) seedlings were used. (**B**) Anti-GFP immunoblot of trypsinized chloroplasts from *Arabidopsis *plants either untreated or treated with 150 mM NaCl. Control immunoblots were probed with antibodies chloroplast proteins RuBisCo and D1; and to cytosolic UGPase. Each lane represents equal amounts of chloroplasts.

To verify the expression levels of *AtTSPO *during salt stress, total protein from each lines was immunoblotted with antibodies to GFP (Figure 8A). In all cases, *At*TSPO:GFP protein was found to increase significantly after 24h of salt treatment. Accumulation of *At*TSPO:GFP was dependent on the presence of *At*TSPO because empty vector controls using CaMV or Ubiquitin 10 [42] promoters to drive the expression of GFP did not change in response to salt stress (Figure 8A and not shown). These results indicate that *At*TSPO accumulation is regulated at the transcriptional, post-transcriptional and post-translational levels.

To confirm the location of *At*TSPO we performed protease protection assays on isolated chloroplasts from OxM1TSPO:eGFP lines that were grown with or without 150 mM NaCl treatment. Following chloroplast isolation and protease protection, equal quantities of chloroplasts were subjected to immunoblotting with antibodies to GFP. *At*TSPO was detected in chloroplast fractions near its predicted monomeric molecular mass (Additional file 4 and 5) in plants treated 18 hours with 150 mM NaCl, but not in untreated plants (Figure 8B). Chloroplasts prepared from OxTSPO:eGFP lines occasionally displayed a lower molecular mass band that is approximately the mass of GFP. This band probably results from proteolysis between *At*TSPO and the GFP tag during sample preparation, although we cannot rule out other possibilities since we do not have an antibody to *At*TSPO protein itself. Antibodies to RuBisCo and D1 confirmed the integrity and presence of chloroplasts following protease protection. Antibodies to the cytosolic protein UGPase also verified these fractions were free of cytoplasmic contamination (Additional file 5). These data together with confocal microscopy indicate that the region between the M1 and M21 is important for targeting *At*TSPO to chloroplasts during salt stress. Since *At*TSPO was protected from trypsin digestion (Figure 8B), *AtTSPO *may be integral to the chloroplast outer envelope.

## Discussion

The localization of TSPO in both chloroplasts and mitochondria is consistent with its role in porphyrin trafficking. Plant TSPO has been proposed to participate in the interaction between plastid and mitochondrial tetrapyrrole biosynthetic pathways [19]. In higher plants, tetrapyrroles are synthesized almost exclusively in plastids, with the exception of the two last steps of heme synthesis that may occur in both chloroplasts and mitochondria. If *At*TSPO is involved in tetrapyrrole transport [19], it is reasonable to assume that *At*TSPO may translocate tetrapyrrole intermediates across organellar membranes, explaining why plants would need chloroplastic and mitochondrial isoforms of TSPO.

Consistent with this hypothesis, the *At*TSPO protein is longer than its mammalian and bacterial counterparts. The targeting determinants for chloroplasts and mitochondria are usually located at the N-terminus of the protein; therefore, a fusion protein with GFP fused to the C-terminus of TSPO was made. Using this strategy we demonstrated that *At*TSPO had different sub-cellular localization patterns depending on the translational start codon, tissue type or the abiotic stress to which the plant was subjected. Placement of the GFP fusion on either the N- or C-terminus of TSPO probably explains the inconsistencies in previous studies [19,21,23] compared to those presented here. The construct used by Guillaumot [23] had the YFP fused to the N-terminus of *At*TSPO, which could potentially mask the transit peptide that targets TSPO to chloroplasts and mitochondria. Indeed, a similar case was observed recently for the *Arabidopsis *HEMERA protein (HMR). When HMR had CFP on its C-terminus, it was localized exclusively in chloroplasts, however, fusion of HMR to the C-terminus of YFP (YFP-HMR) was localized to the nucleus and cytoplasm but not chloroplasts [43].

Salt-stress of *Arabidopsis *results in movement of ER-localized *At*M1TSPO:eGFP to the chloroplast. We also demonstrated that the different start codons within the TSPO N-terminal extension could target the TSPO protein to different organelles. Other plant proteins such as MDAR (Arabidopsis Monodehydroascorbate Reductase) and tRNA nucleotidyltransferase [44,45] are also known to be targeted to different organelles owing to alternative transcriptionsal start sites. Thus, it is tempting to speculate that, cloroplastic *At*TSPO may protect the chloroplast from salt stress damage and the mitochondrial *At*TSPO may normally import chloroplast-synthesized porphyrins into the mitochondria. Alterations in the sub-cellular localization of TSPO have been observed in mammals. The mammalian TSPO localizes to the mitochondrial outer membrane but during fast cell proliferation, such as metastatic processes, it relocates to the nuclear membrane, suggesting developmental control of its sub-cellular localization [46].

Our results suggest the existence of a chloroplast targeting region in *At*TSPO that operates during salt stress. Constructs lacking the N-terminus of *At*TSPO (*At*M2TSPO:eGFP and *At*M3TSPO:eGFP) are not able to be target to this organelle. These results suggest that the first twenty aminoacids of *At*TSPO may be part of the chloroplast targeting peptide of this protein. Further experiments should be conducted to precisely characterize this chloroplast targeting determinant.

TSPO localization in plant cells is complex, involving a relocation of the protein from ER and vesicles to chloroplasts during salt-stress. In recent years, several new mechanisms for import of proteins into chloroplasts have been proposed. For example, it is hypothesized that close contacts between the envelopes of chloroplasts, mitochondria and other organelle membranes could allow protein movement between them [47]. Such fusions have been observed between the mitochondria and ER [48], where it was suggested that vesicle associated membrane protein 1 (VAMP-1) might be involved in the docking of mitochondria to target membranes [49]. This, in turn, could facilitate a re-localization of proteins from mitochondria to other compartments. The recent discoveries of close intracellular membrane contacts in plants, namely between chloroplasts and the ER [50], as well as between mitochondria and the nucleus [51], corroborates this hypothesis. At the present moment it is not clear which pathway is used during *At*TSPO relocation during salt-stress. However our data indicate that *At*TSPO changes its localization during stress, and that it is also possible that the mitochondrial isoform observed previously by Frank *et al*. [22]*in P. patens *and by Lindenman *et al*. [19] in *D. lanata *and *Arabidopsis *could be generated in *Arabidopsis *by the use of alternative translation start codons.

Transcriptional levels of *AtTSPO *in wild-type *Arabidopsis *plants increase in response to salt, mannitol, ABA and paraquat. The promoter region of *At*TSPO was also found to be sufficient for salt stress transcriptional response. The induction of *AtTSPO *by salt stress was also observed when constitutive promoters (35S and UBI10) were used to express *AtTSPO*, suggesting that the induction of *At*TSPO occurs at both transcriptional and post-transcriptional levels.

*At*TSPO over-expression lines have decreased levels of stress response genes (*ERD10*, *DREB2A *and *RAB18*), while *tspo-1 *mutants over express these genes. This suggests that *AtTSPO *expression and/or function is necessary for the proper regulation of these genes during stress conditions. These results also imply that *At*TSPO is important for stress adaptation in *Arabidopsis*, and this idea is consistent with results from *P. patens *[22]. Since *Rhodobacter *TSPO is a negative regulator of photosynthetic genes [17], it is possible that *At*TSPO operates similarly in regulating stress responsive genes in plants.

The precise function of *At*TSPO in tetrapyrrole transport during salt stress remains to be established. There are, however, many reports suggesting that alterations in tetrapyrrole flow can be involved in salt tolerance. Exogenous 5-Aminolevulinate (ALA) can improve salt tolerance in higher plants [51-56]. It has been also shown that transgenic *Arabidopsis*, tobacco and rice that overproduce ALA have improved salt tolerance [57,58]. Abdelkader *et al*. [59] assumed that high salt stress inhibited chlorophyll accumulation mainly by reducing the rate of porphyrin formation, and Zhang *et al*. [58] showed that salt stress caused a significant decrease in heme content. Thus in higher plants, ALA and tetrapyrrole synthesis is sensitive to salt stress.

Additionally, we demonstrated that *At*TSPO is important for tetrapyrrole flux and/or metabolism. The herbicide Norflurazon, a non-competitive inhibitor of phytoene desaturase, [31-33] and the neurotoxin Gabaculine, which inhibits tetrapyrrole biosynthesis by blocking glutamate-1-semi aldehyde aminotransferase activity [29,30] were used in this study to decrease the flux through the tetrapyrrole biosynthesis pathways. Our data showed that mutations in tetrapyrrole biosynthesis genes and the application of these two different drugs that decrease flux of tetrapyrrole intermediates affect *AtTSPO *expression. All mutations tested that inhibit the synthesis of ALA increase *AtTSPO *mRNA steady-state levels. The same was observed when the formation of ALA is inhibited by the norflurazon and the gabaculine. The only mutant tested with decreased *AtTSPO *expression is *crd1*, which accumulates Mg-Protoporphyrin monomethyl ester and this accumulation does not affect the inhibition of ALA synthesis [40]. Finally, it is possible that *At*TSPO could be involved in the partitioning of different tetrapyrrolic signal molecules within plant cells. The steady state levels of several light-regulated mRNAs of tetrapyrrole metabolism genes are down-regulated in the *tspo-1 *mutant, suggesting that, *At*TSPO could act as a regulator of tetrapyrrole biosynthesis similar to its bacterial counterpart [16].

## Conclusions

TSPO has been shown to transport a number of small molecules in multiple organisms, however its function in plants is not known. Here we demonstrate that *Arabidopsis TSPO *is regulated at the transcriptional, post-transcriptional and post-translational levels in response to abiotic stress conditions such as salt stress. Our results suggest that *At*TSPO can localize to ER and mitochondria, but when plants are salt stressed *AtTSPO *is found in chloroplasts. Also our data suggest that under normal conditions *AtTSPO *may be important for the import of chloroplastic synthesized heme into the mitochondria. However, targeting *AtTSPO *to the chloroplast during salt stress may protect chloroplasts from damage. In addition, tetrapyrrole intermediates has been suggested to operate in the chloroplast-to-nucleus retrograde signaling [35,60]. It is possible that *At*TSPO could be involved in the partitioning of different tetrapyrrole signal molecules within plant cells depending on environmental conditions. *At*TSPO may play a role in re-directing tetrapyrrole intermediates during salt stress or under conditions where tetrapyrrole metabolism is compromised. This is suggested by our finding that mutation or inhibition of the tetrapyrrole biosynthesis pathway increases *AtTSPO *expression. At the same time, *At*TSPO may directly contribute to the detoxification of highly reactive porphyrins in the cytoplasm. We are currently investigating these possibilities.

## Methods

### Plant material and growth conditions

*Arabidopsis thaliana *seeds ecotype *Col-0 *were surface sterilized and plated on MS_1/2 _medium [61] with or without 50 mM kanamycin. Seedlings were maintained for three days at 4°C and than grown under 16/8 hours light/dark cycles at 23°C in growth chambers. Root length measurements were conducted using plants grown on vertically oriented in standard conditions for 10 days. For abiotic stress treatment, 150 mM NaCl, 250 mM mannitol, 1 μM ABA (Sigma; St Louis, MO) or 0.2 μM paraquat was added to MS_1/2 _agar plates, and the 5-day-old seedlings were incubated under normal growth condition. For Norflurazon or Gabaculine experiments seeds were plated on MS_1/2 _containing 1 or 2% sucrose with or without 5 μM norflurazon (Sandoz Pharmaceuticals; Vienna, Austria) or 50 μM of gabaculine (Sigma, USA). All experiments were repeated three times independently and the average was calculated.

### RNA extraction and qRT-PCR analysis

Total RNA was isolated using Spectrum™ Plant Total RNA Kit (Sigma #STRN250-1KT), according to manufacturer's instructions. One microgram of total RNA was added to each cDNA synthesis reaction using the First Strand cDNA Synthesis Kit (#K1611). For qRT-PCR, DNA amplification was performed in the presence of SYBR^® ^Green qPCR Detection (Invitrogen) in a MyIQ™ Single Color Real-Time PCR Detection System (BioRad), using the primer pairs at table 1. The cycle use was: 95 C, 1 min and 30 sec; 40 × (95 C, 10 sec; 60 C, 1 min); 95 C, 1 min; 60 C, 1 min and 81 × (60 C, 10 sec). The relative mRNA levels were determined by normalizing the PCR threshold cycle number with *Actin *and *18S RNA*. All experiments were repeated three times independently and the average was calculated.

**Table 1:** Primers used for quantitative real time PCR (qRT-PCR)

PRIMER NAME FOR qPCR	SEQUENCE
TSPO FWD	ACAAAGGAAAACGCGATCAAA
TSPO RVS	ACTTGAGACCACGTTTCGCC

GUN1 FWD	GCGATTCTGAATGCTTGCAG

GUN1 RVS	AGGAGCCATACATTCTCTCT

GUN2 FWD	AGACTCCAATTTCCCAACTT

GUN2 RVS	TTACCAGGACGTGTTGGTTC

GUN4 FWD	GAAACCGCGACCATATTCGAC

GUN4 RVS	CGGCTTCTCCGGATATCTGAA

GUN5 FWD	CATCCACTTGCTCCAACCATG

GUN5 RVS	CCGACAACCGTTGCATCTTT

HEMA1 FWD	GCTTCCGCAGTCTTCAAACG

HEMA1 RVS	CCAGCGCCAATTACACACATC

HEMA2 FWD	AGCTCCTGCACGGTCCAAT

HEMA2 RVS	TGCTATCGTTCCCATCGCAT

FC1 FWD	ATACCAGAGTCGTGTTGGCCC

FC1 RVS	TCATCGGTGTATGGCTTCAGC

FC2 FWD	TGGTGCTATGGCTGTCTCAAAC

FC2 RVS	AGCGGAACTAACGACTGTCGA

CAO FWD	TGATGAGCCACCTGCACCTAT

CAO RVS	AAGTAAACCGTGTTCCACCGG

PPO FWD	GCTTCTTCCGTCGTTTTCGAA

PPO RVS	TTGAAGATCCGACGGTTGGTC

DREB2A FWD	CAGGCTTAAATCAGGACCGG

DREB2A RVS	ATGAACCGTTGGCAACACTG

ERD10 FWD	CACCGTTCCAGAGCAGGAGA

ERD10 RVS	GCCGATGATTCCTCTGTTGC

RAB18 FWD	AAGGAGAAGTTGCCAGGTCATC

RAB18 RVS	CATCGCTTGAGCTTGACCAG

ACTIN 2/8 FWD	TCTTGTTCCAGCCCTCGTTT

ACTIN 2/8 RVS	TCTCGTGGATTCCAGCAGCT

18S RNA FWD	TATAGGACTCCGCTGGCACC

*18S *RNA RVS	CCCGGAACCCAAAAACTTTG

### Verification of TSPO knock-out

The *tspo-1 *T-DNA mutant, SALK_135023, was obtained from the Salk collection [24]. Homozygous mutants were isolated by PCR-based genotyping using gene specific PCR primers *At*TSPO-LP and *At*TSPO-BP together with LBa1 (Table 2). Only homozygous lines were used for the phenotypic investigation.

**Table 2:** Primers used for cloning and genotyping

PRIMER NAME FOR GENOTYPING AND CLONING	SEQUENCE
**AtTSPO LP**	agagcaaatcgcatcagcgtc

**AtTSPO RP**	ggaacgtaaccggatcccaaa

**LBa1**	tggttcacgtagtgggccatcg

**TSPO NT1**	aaaaagcaggctccatggattctcaggaca

**TSPO NT2**	aaaaagcaggctccatggccgagacagagagg

**TSPO NT3**	aaaaagcaggctccatggcgaaacgtggtctc

**TSPO CT1**	agaaagctgggtccgcgacagcaagctttaca

**TSPO CT80**	agaaagctgggtcggacttagctcgattcccgta

### Construction of AtTSPO GUS Fusion Vector and GUS Assay

The 437 bp upstream of the translational star site of the *AtTSPO *gene (*At*2g47770) was translational fused into *uidA *gene in pKGWFS7 vector by Gateway^® ^(Invitrogen™) [62] and introduced into *Arabidopsis *via *Agrobacterium*-mediated transformation [63]. For cloning primers and constructs information see Tables 2 and 3, respectively. For histochemical GUS expression plant samples were soaked at 37°C for 16 hours in GUS assay solution (1 mm 5-bromo-4-chloro-3-indolylglucronide, 0.5 mm K_3_Fe(CN)_6_, 0.5 mm K_4_Fe(CN)_6_, 0.3% (v/v) Triton X-100, 20% (v/v) methanol, and 50 mm inorganic phosphate-buffered saline). The reaction was further conducted at 37°C in the dark for a maximum of 16 hours.

**Table 3:** Constructs information

CONSTRUCT NAME	BINARY VECTOR	RESISTANCE IN PLANT
UBQ10mCITRINE	pB7m34GW	basta

UBQ10M1TSPOmCITRINE	pB7m34GW	basta

UBQ10M2TSPOmCITRINE	pB7m34GW	basta

UBQ10M3TSPOmCITRINE	pB7m34GW	basta

OxeGFP	pK7FWG2	kanamycin

OxMITSPO:eGFP	pK7FWG2	kanamycin

OxM2TSPO:eGFP	pK7FWG2	kanamycin

OxM3TSPO:eGFP	pK7FWG2	kanamycin

AtTSPO-437::GUS	pKGWFS7	kanamycin

### Subcellular localization of AtTSPO fusion proteins

For the GFP fusion constructs, clones containing the coding region of *AtTSPO *as well as fusions starting at methionine 21 and 42 were generated and cloned into pK7FWG2 [62] (Table 3) according to the manufacturer's instructions (Invitrogen, CA, USA). Primers used were: *At*TSPO M1: TSPO NT1 and TSPO CT1; *At*TSPO M2: TSPO NT2 and TSPO CT1; *At*TSPO M3: TSPO NT3 and TSPO CT1; *At*TSPO 80aa: TSPO NT1 and TSPO CT80 (Table 2). *Arabidopsis thaliana *was observed in a confocal laser scanning microscope Leica DM IRE2 (Leica microsystems). For the mitochondrial-specific staining, *Arabidopsis *seedlings were incubated in MitoTracker^® ^Red CMXRos (Invitrogen, #M7512) according to manufactures instructions. Excitation and emission wavelengths were 488 and 505-530 nm (BP 505-530 filter) for GFP and, 543 and 560-615 nm (BP 560-615 filter) for MitoTracker^® ^respectively. All images were processed on Leica DM IRE2 Image Browser program (Leica microsystems).

### Determination of chlorophyll contents

Seedlings at 10 days after germination were weighted, frozen in liquid nitrogen, and ground in 80% (v/v) acetone. Ground tissue was centrifuged at 2,000 g for 5 min to pellet any insoluble material. The absorbance of the extracted chlorophyll at 645 and 663 nm was then determined. Chlorophyll (a and b) contents of the samples were determined according to Lichtenthaler [64].

### Chloroplast Isolation

Isolation of chloroplasts from plate-grown *Arabidopsis *seedlings was performed as described previously [65]. Final resuspension of chloroplast was in buffer (330 mM sorbitol, 50 mM HEPES-KOH, pH 8.0) at a concentration of 1 mg chlorophyll ml^-1^.

### Immunoblotting

Total protein was extracted from 10-day-old seedlings by adding protein extraction buffer (50 mM HEPES pH 7.9, 300 mM Sucrose, 150 mM NaCl, 10 mM Potassium acetate, protease inhibitors cocktail - Roche, 1% (w/v) Triton, 1 mM DTT). Ground tissue was centrifuged at 5,000 × g for 5 min to pellet the tissue and proteins were quantify by Bradford assay [66]. Samples were boiled for 5 min in 250 mM Tris-HCl, pH 6.8, 10% (w/v) SDS, 30% (v/v) glycerol, 5% (v/v) β- mercaptoethanol and 0.02% (w/v) bromophenol blue. SDS-PAGE was performed using standard procedures. Chloroplast protein samples were normalized loaded by equal amounts of total chlorophyll. Following SDS-PAGE, the separated proteins were transferred to a polyvinylidene difluoride membrane (Bio-Rad). For immunodetection, membranes were incubated with antibody against GFP (ROCHE, #11814460001), UGPase (AGRISERA, #AS05086), RuBisCo (AGRISERA, #AS03037) and D1(AGRISERA, #AS05084). With the exception of GFP detection that uses mouse secondary antibody, all the immunoreactive proteins were detected by using rabbit secondary antibody. The immunoreaction was detected by chemiluminescence kit (Thermo Scientific, #34076) according to manufacturer's instructions.

## Abbreviations

ABA: Abscisic acid; ALA: 5-Aminolevulinate; CFP: Cyan fluorescent protein; D1: photosystem II reaction center D1 protein; HMR: Hemera protein; GFP: Green fluorescent protein; GUS: β-Glucuronidase; Mg-Proto IX: Mg-Protoporphyrin IX; MV: Methyl viologen; ROS: Reactive oxygen species; RUBISCO: Ribulose-1,5-biphosphate carboxylase; TSPO: 18 kDa Translocator protein; UGPase: UDP-glucose pyrophosphorylase; VAMP: Vesicle associated membrane protein; YFP: Yellow fluorescent protein.

## Authors' contributions

EBP, JC and GSM conceived and designed the experiments. EBP performed all the experiments, analyzed the data and wrote the paper. YJ helped in the confocal microscopy analyses. BJSCO helped in the fractionation experiment. LRA and JGU gave technical support. JC and GSM were project supervisors, participated in the discussion of all experiments from the project and preparation of the manuscript. All authors read and approved the final manuscript.

## Supplementary Material

Additional file 1 **Schematic representation of tetrapyrrole biosyntheses pathway in plants showing genes analyzed in this study**. In blue, are the genes already described for each step in the pathway. The enzymes that correspond to these genes names and the AGI code are: HEMA1 (Glutamyl-tRNA reductase 1, *At*1g58290); HEMA2 (Glutamyl-tRNA reductase 2, *At*1g09940); HEMA3(Glutamyl-tRNA reductase 3, *At*2g31250); FLU (Regulator of ALA synthesis, *At*3g14110); LIN2 (Coproporphyrinogen oxidase 1, *At*1g03475); GUN2 (Heme oxygenase 1, *At*2g26670); GUN3 (Phytochromobilin synthase, *At*3g09150); GUN4 (Regulator of Mg-porphyrin synthesis, *At*3g59400); GUN5 (Mg-chelatase subunit H, *At*5g13630); CHLI (Mg-chelatase subunit I, *At*4g18480 and *At*5g45930); CHLD (Mg-chelatase subunit D, *At*1g08520); CHLM (Mg-Protoporphyrin IX methyltransferase, *At*4g25080); CRD1 (Mg-Protoporphyrin IX monomethylester cyclase, *At*3g56940); FC1 (Ferrochelatase 1, *At*5g26030); FC2 (Ferrochelatase 2, *At*2g30390). Click here for file

Additional file 2 **Alignment of TSPO sequences from different organisms**. ClustalW sequence alignment of TSPO proteins from *Rhodobacter sphaeroides *(AF195122.1), *Rattus norvegicus *(J05122) and *Arabidopsis *TSPO (*At*TSPO - *At*2g47770). The numbers in the left side represent the amino acid position from the primary protein. In the consensus line the conserved aminoacids are highlighted as (*), and as (.) when one conserve position is observed. M1, M21 and M42 *At*TSPO isoforms are highlighted. The black arrow represents the first 80 aminoacids (*At*TSPO80aa) of *Arabidopsis *TSPO. Click here for file

Additional file 3 ***At*M42TSPO:eGFP co-localizes with mitotracker in *Arabidopsis thaliana***. *At*M42TSPO:eGFP 5-day-old seedlings transgenic lines (A-C) were incubated with mitotracker to identify mitochondria. (A) Image from GFP channel is shown in green. (B) Image from mitotracker channel is shown in red. (C) Merge between GFP and mitotracker channels shown in yellow. Scale bar = 50 μM. Click here for file

Additional file 4 **Immunoblot showing that *At*TSPO:eGFP accumulates during salt stress**. Immunoblot analysis of protein level in all three isoforms of Ox*At*TSPO:eGFP (OxM1TSPO:eGFP, OxM21TSPO:eGFP and OxM42TSPO:eGFP) during salt stress show accumulation of the protein. As a control wild-type plants and plants over-expressing GFP (OxeGFP) were used. Anti-UGPase and Red-ponceau staining were used as loading controls. Equal amounts of total protein were loaded. Click here for file

Additional file 5 **Immunoblot of chloroplasts prepared from OxTSPO:eGFP plants**. *Arabidopsis *chloroplasts were prepared from 10-days-old seedlings either untreated or treated with 150 mM NaCl and immunoblotted with antibodies to GFP, RuBisCo, D1 and UGPase. Equal amounts of OxM1TSPO:eGFP chloroplast protein samples were loaded in each lane. (TP) Total Protein; (Chl) Chloroplast protein; PP (Protease Protection treatment). Click here for file
