# Supplementary figures and images for: The Arabidopsis translocator protein (AtTSPO) is regulated at multiple levels in response to salt stress and perturbations in tetrapyrrole metabolism
Source: BMC Plant Biol. 2011 Jun 20;11:108. doi: 10.1186/1471-2229-11-108 (PMC3141639; doi:10.1186/1471-2229-11-108)

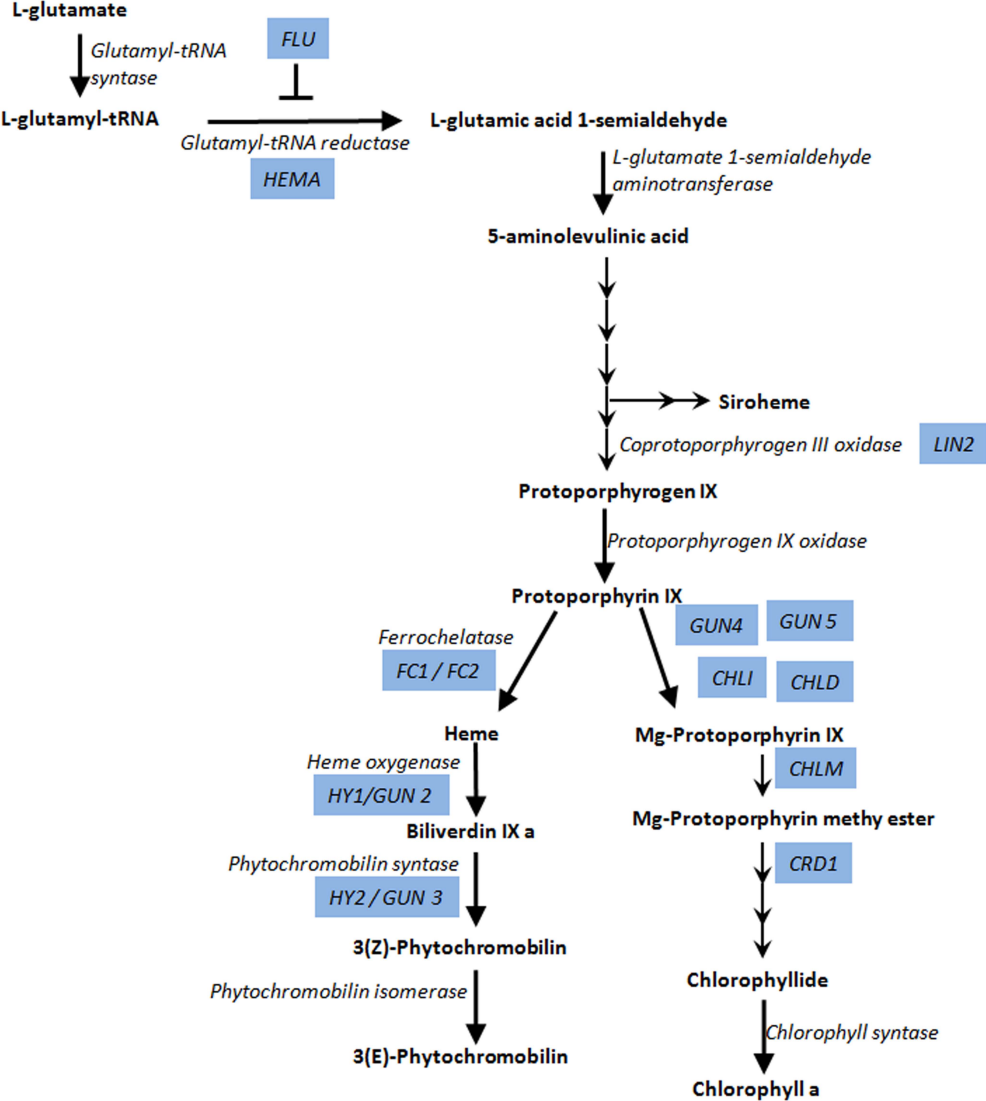

Supplement: Additional file 1 — Schematic representation of tetrapyrrole biosyntheses pathway in plants showing genes analyzed in this study. In blue, are the genes already described for each step in the pathway. The enzymes that correspond to these genes names and the AGI code are: HEMA1 (Glutamyl-tRNA reductase 1, At1g58290); HEMA2 (Glutamyl-tRNA reductase 2, At1g09940); HEMA3(Glutamyl-tRNA reductase 3, At2g31250); FLU (Regulator of ALA synthesis, At3g14110); LIN2 (Coproporphyrinogen oxidase 1, At1g03475); GUN2 (Heme oxygenase 1, At2g26670); GUN3 (Phytochromobilin synthase, At3g09150); GUN4 (Regulator of Mg-porphyrin synthesis, At3g59400); GUN5 (Mg-chelatase subunit H, At5g13630); CHLI (Mg-chelatase subunit I, At4g18480 and At5g45930); CHLD (Mg-chelatase subunit D, At1g08520); CHLM (Mg-Protoporphyrin IX methyltransferase, At4g25080); CRD1 (Mg-Protoporphyrin IX monomethylester cyclase, At3g56940); FC1 (Ferrochelatase 1, At5g26030); FC2 (Ferrochelatase 2, At2g30390). [file 1471-2229-11-108-S1.PDF]

# OxM42TSPO:eGFP

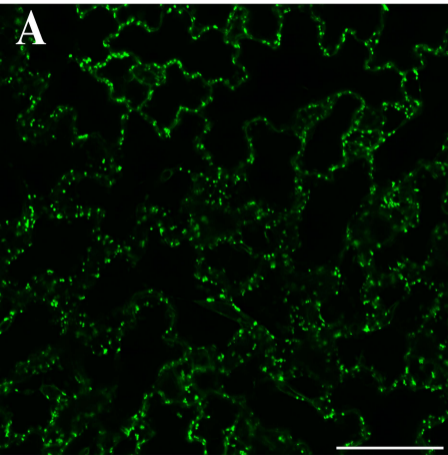

**GFP**

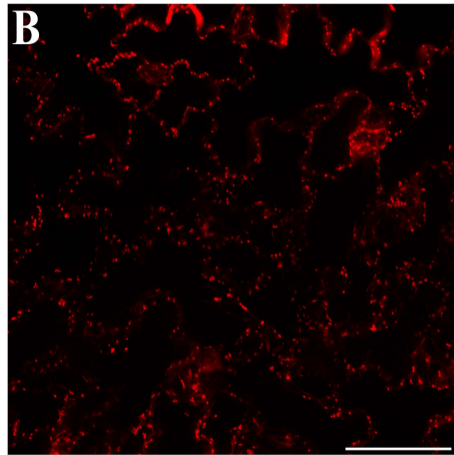

**MITOTRACKER**

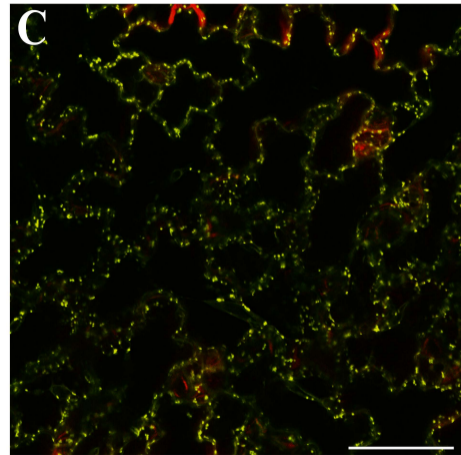

**MERGE**

Supplement: Additional file 3 — AtM42TSPO:eGFP co-localizes with mitotracker in Arabidopsis thaliana. AtM42TSPO:eGFP 5-day-old seedlings transgenic lines (A-C) were incubated with mitotracker to identify mitochondria. (A) Image from GFP channel is shown in green. (B) Image from mitotracker channel is shown in red. (C) Merge between GFP and mitotracker channels shown in yellow. Scale bar = 50 μM. [file 1471-2229-11-108-S3.PDF]

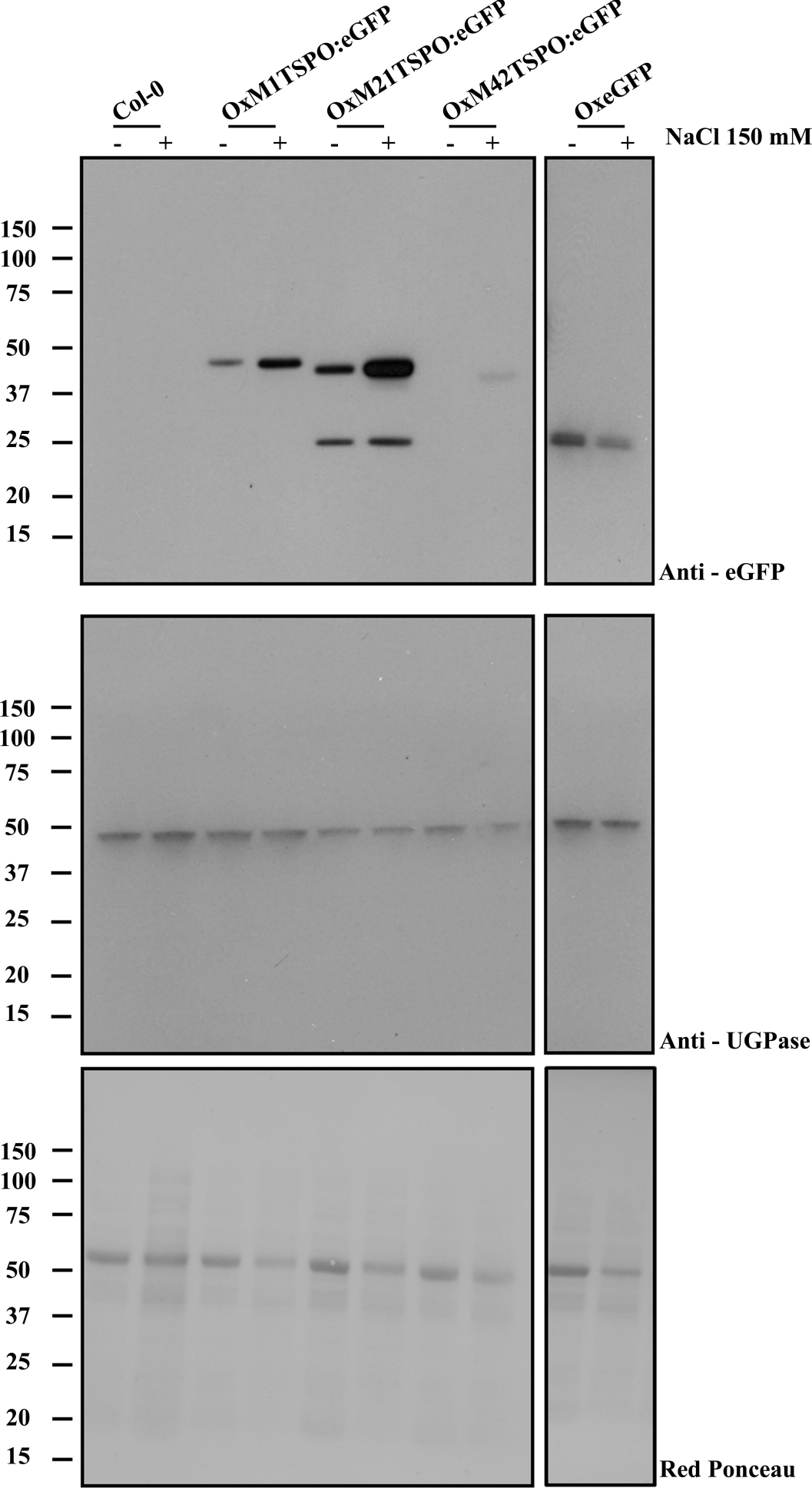

Supplement: Additional file 4 — Immunoblot showing that AtTSPO:eGFP accumulates during salt stress. Immunoblot analysis of protein level in all three isoforms of OxAtTSPO:eGFP (OxM1TSPO:eGFP, OxM21TSPO:eGFP and OxM42TSPO:eGFP) during salt stress show accumulation of the protein. As a control wild-type plants and plants over-expressing GFP (OxeGFP) were used. Anti-UGPase and Red-ponceau staining were used as loading controls. Equal amounts of total protein were loaded. [file 1471-2229-11-108-S4.PDF]

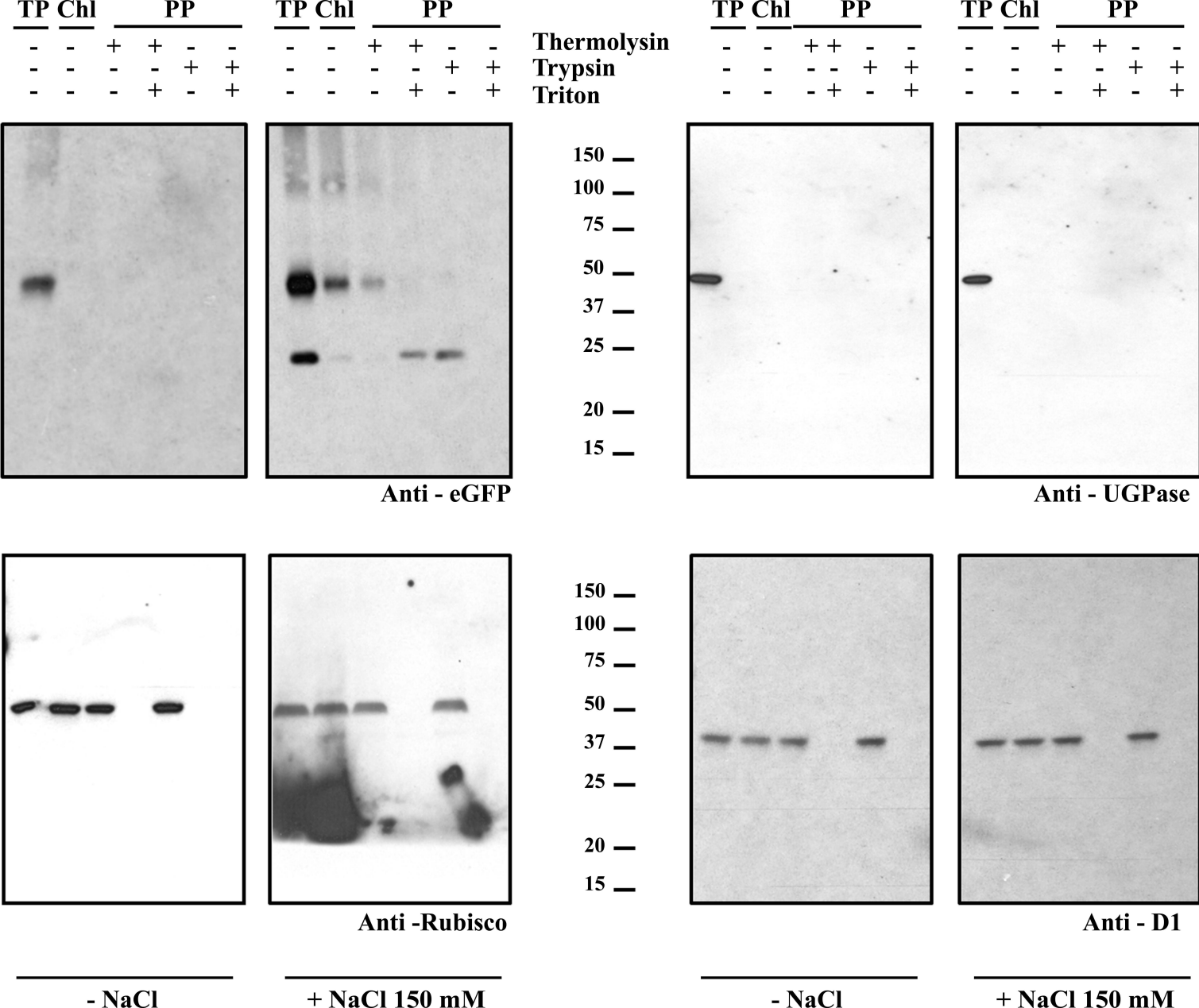

Supplement: Additional file 5 — Immunoblot of chloroplasts prepared from OxTSPO:eGFP plants. Arabidopsis chloroplasts were prepared from 10-days-old seedlings either untreated or treated with 150 mM NaCl and immunoblotted with antibodies to GFP, RuBisCo, D1 and UGPase. Equal amounts of OxM1TSPO:eGFP chloroplast protein samples were loaded in each lane. (TP) Total Protein; (Chl) Chloroplast protein; PP (Protease Protection treatment). [file 1471-2229-11-108-S5.PDF]
