# Supplementary material for: The Arabidopsis translocator protein (AtTSPO) is regulated at multiple levels in response to salt stress and perturbations in tetrapyrrole metabolism
Source: BMC Plant Biol. 2011 Jun 20;11:108. doi: 10.1186/1471-2229-11-108 (PMC3141639; doi:10.1186/1471-2229-11-108)
Supplement: Additional file 2 — Alignment of TSPO sequences from different organisms. ClustalW sequence alignment of TSPO proteins from Rhodobacter sphaeroides (AF195122.1), Rattus norvegicus (J05122) and Arabidopsis TSPO (AtTSPO - At2g47770). The numbers in the left side represent the amino acid position from the primary protein. In the consensus line the conserved aminoacids are highlighted as (*), and as (.) when one conserve position is observed. M1, M21 and M42 AtTSPO isoforms are highlighted. The black arrow represents the first 80 aminoacids (AtTSPO80aa) of Arabidopsis TSPO. [file 1471-2229-11-108-S2.PDF]

```
R.norvegicus        ----------------------------------------MSQSWVPAVGLTLVPSLGG 19
R.sphaeroides       ---------------------------------------MMNMDWALFLTFLAACGAPA 20
AtTSPO              MDSQDIRYRGGDDRDAATTAMAETERKSADDNKGKRDQKRAMAKRGLKSLTVAVAAPVLV 60
                                                             *       : .  .
                    M1                  M21                  M42

R.norvegicus        FMGAYFVRGEGLRWYASLQKPSWHPPRWTLAPIWGTLYSAMGYGSYIIWKELGGFTEEAM 79
R.sphaeroides       TTGALLKPDE---WYDNLNKPWWNPPRWVFPLAWTSLYFLMSLAAMRVAQLEG-----SG 72
AtTSPO              TLFATYFLGTSDGYGNRAKSSSWIPPLWLLHTTCLASSGLMGLAAWLVWVDGG--FHKKP 118
                       *    .    :    :.. * ** * :     :    *. .:  :    *

R.norvegicus        VPLGLYTGQLALNWAWPPIFFGARQMGWALVDLMLVSGVATATTLAWHRVSPPAARLLYP 139
R.sphaeroides       QALAFYAAQLAFNTLWTPVFFGMKRMATALAVVMVMWLFVAATMWAFFQLDTWAGVLFVP 132
AtTSPO              NALYLYLAQFLLCLVWDPVTFRVGSGVAGLAVWLGQSAALFGCYKAFNEISPVAGNLVKP 178
                     .* :* .*: :   * *: *       .*.  :       .   *: .:.. *. *. *

R.norvegicus        YLAWLAFATMLNYYVWRDNSGRRGGSRLTE 169
R.sphaeroides       YLIWATAATGLNFEAMRLNWNRPEARA--- 159
AtTSPO              CLAWAAFVAAVNVKLAVA------------ 196
                     * * : .: :*
```
